# Power-Dissipation Model for Water Dissociation in Bipolar Membranes: Model Extension to High Current Density and Experimental Validation

**DOI:** 10.3390/membranes16090283

**Published:** 2026-08-26

**Authors:** Mohamed Fadel Anass Ma-el-ainine, Rachid Boukhili, Oumarou Savadogo

**Affiliations:** 1 Laboratory of New Materials for Energy and Electrochemistry, Polytechnique-Montréal, Montreal, QC H3T 1J4, Canada; oumarou.savadogo@polymtl.ca; 2 Research Centre for High Performance Polymer and Composite Systems (CREPEC), Mechanical Engineering Department, Polytechnique-Montréal, Montreal, QC H3T 1J4, Canada; rachid.boukhili@polymtl.ca

**Keywords:** bipolar membrane, water dissociation, reverse bias, electric field, power-dissipation model, quadratic current–voltage law, water-transport limitation, limiting current density, acid–base electrolysis, ion-exchange membrane

## Abstract

Water dissociation (WD) at the internal junction of bipolar membranes (BPMs) is the key process enabling acid/base generation under reverse bias, yet the physical origin of its strong enhancement remains debated. In our previous work, we proposed a power-dissipation model in which WD is enhanced by an intense electric field through local power dissipation by autoprotolysis ions. Here, we experimentally validate this model using three commercial BPMs under acid/base and neutral salt conditions and extend it to high current density by incorporating finite water supply to the BPM junction. Under acid/base conditions, two BPMs showed strong field-dominated quadratic behavior, while a catalyst-containing BPM displayed a predominantly linear response, indicating that heterogeneous interfacial catalysis can mask the purely field-driven signature. In neutral $Na_{2}SO_{4}$, all three BPMs exhibited excellent quadratic fits, confirming that the predicted $J_{WD}\propto U_{j}^{2}$ behavior is robust. The fitted prefactor varied with membrane type and electrolyte configuration, reflecting differences in hydration, junction thickness, transport behavior, and catalysis effect. The model was extended at higher current density to include finite diffusive water supply to the junction. The resulting saturation law links the intrinsic quadratic WD current $J_{WD}$ to a water-transport-limited current $J_{lim,2}$, and predicts an inflection point at $J_{WD,inf}\;=\;0.25\;J_{lim,2}$.

## 1. Introduction

**Figure 1:**
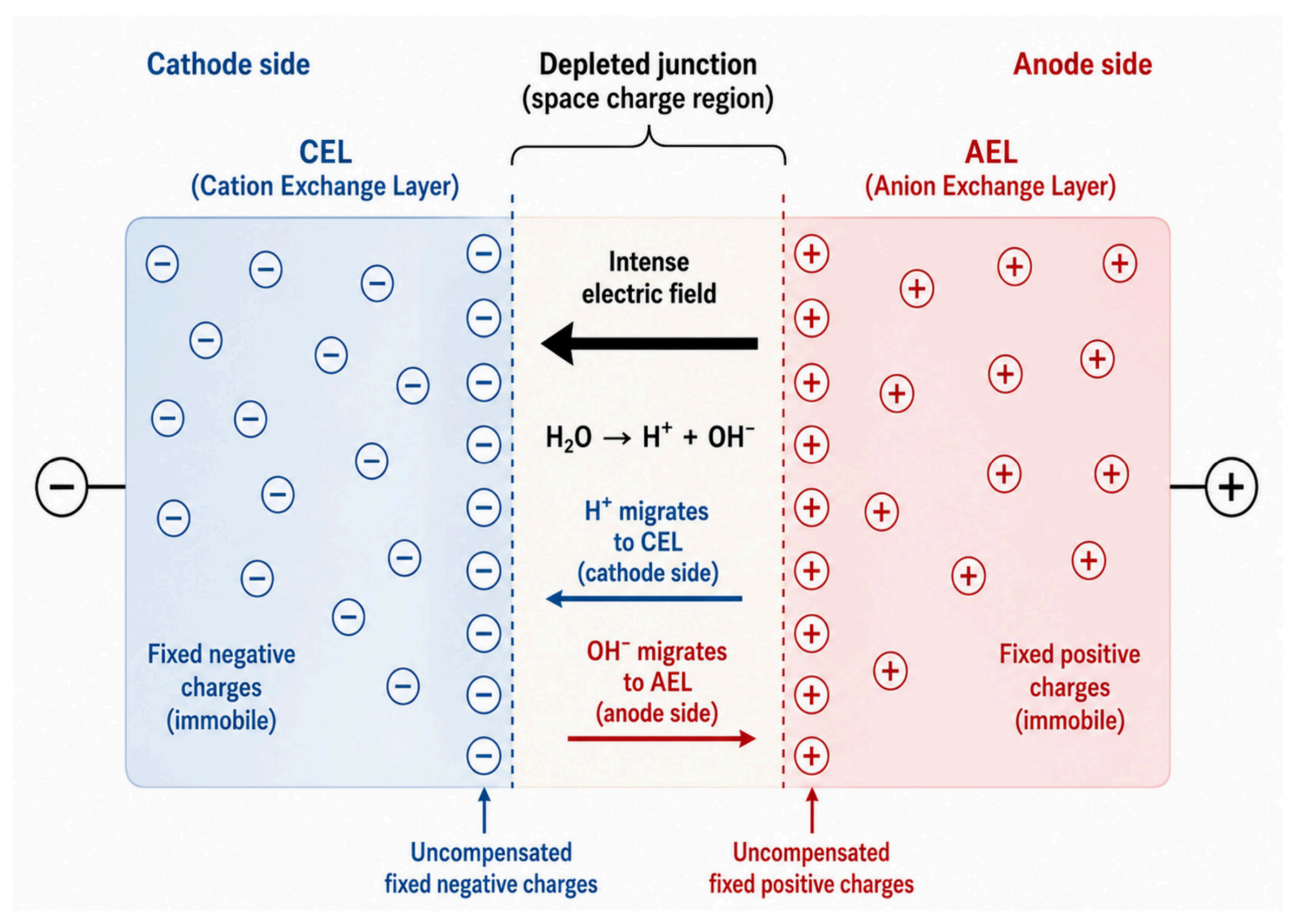
Principle of water dissociation in a bipolar membrane junction under reverse bias.

Bipolar membranes (BPMs) are increasingly used in electrochemical and electromembrane processes due to their ability to sustain pH gradients while maintaining ionic current through internal water dissociation [1,2,3,4,5]. By combining a cation exchange layer (CEL) and an anion exchange layer (AEL), a BPM creates a narrow interfacial junction where water can be dissociated into $H^{+}$ and $OH^{-}$ under reverse bias [1,2,6]. The generated protons and hydroxide ions migrate through the CEL and AEL, respectively, enabling acid/base generation and pH control without direct chemical addition (Figure 1). This unique function has made BPMs important in electrodialysis for acid and base production [7], pH regulation [8], seawater desalination [9,10], carbon dioxide capture and transformation [11,12], electrosynthesis [13], fuel cells [14], and emerging water-electrolysis architectures [15]. In acid–alkaline water electrolysis, BPMs are particularly attractive because they can allow hydrogen evolution to occur in acidic media while oxygen evolution proceeds in alkaline media, thereby expanding the range of usable electrode materials and reaction environments [16,17,18,19]. In all of these applications, the performance of the device ultimately depends on how efficiently the BPM junction can sustain water dissociation at technologically relevant current densities [20].

Despite this growing technological relevance, BPMs rely on a phenomenon that remains unresolved: the dramatic acceleration of water dissociation (WD) at the internal junction. In bulk water, autoprotolysis is slow, with a dissociation rate constant of $k_{d}\;\approx \;2\;\times \;10^{-5}\;s^{-1}$ [21]. Such a rate is far too small to account for the current densities commonly measured in operating BPMs, which exceed hundreds of $mA\;cm^{-2}.$ Thus, the effective WD rate in the junction must be enhanced by several orders of magnitude, typically approaching or exceeding $10^{7}$ relative to the thermal value [17,22]. This quantitative mismatch defines the fundamental mechanistic problem: what physical or chemical process in a reverse-biased BPM junction can increase the effective WD rate sufficiently to account for the observed current densities?

Two broad classes of mechanisms have traditionally been invoked to answer this question. The first attributes WD enhancement to catalytic protonation–deprotonation reactions involving fixed membrane groups with water molecules, which mediate proton transfer and lower the kinetic barrier for $H^{+}$ and $OH^{-}$ generation [23,24]. This mechanism is important in many BPMs, especially those containing weak acid/base groups or intentionally added catalysts [25]. However, the predicted rates are often too low unless the acid–base properties of the functional groups are optimized; for example, with basic groups having a $pK_{b}$ close to the neutral water range [22]. This requirement is restrictive, because commercial BPMs contain different fixed groups that do not necessarily have such ideal acid–base properties. Moreover, modifying fixed groups to optimize proton-transfer catalysis may compromise other membrane properties, including ionic conductivity and permselectivity.

The second major class of explanations is based on the role of the electric field. Under reverse bias, i.e., when the CEL faces the cathode and the AEL faces the anode, mobile ions are progressively removed from the CEL/AEL interface. As the junction becomes depleted (Figure 1), the fixed charges of the two ion-exchange layers become insufficiently compensated by mobile counter-ions, and a strong electric field is established across a narrow space charge region (SCR) of a few nanometers [26,27,28]. The Second Wien Effect (SWE) has historically been used to describe this field-enhanced dissociation. Although it captures the intuitive importance of electric fields, its formulation as given by Onsager often underpredicts the magnitude of $k_{d}$ required experimentally, when directly applied to BPM-relevant fields [22]. Attempts to improve the prediction frequently rely on uncertain assumptions about the local dielectric constant in the confined and charged BPM junction [22,29]. Reducing the dielectric constant can increase the predicted enhancement, but it also makes the result extremely sensitive and can lead to unstable or divergent estimates [29,30]. Hybrid models, such as the field-assisted chemical reaction model (CRM) [31], attempt to combine catalytic proton-transfer reactions with electric-field effects, but they often introduce poorly constrained parameters [6,32]. Consequently, a simple and physically transparent link between the local field and the observed WD current remains needed.

This mechanistic uncertainty between catalytic and field-driven mechanisms has direct consequences for BPM design. If proton-transfer catalysis dominates, high-performance BPMs should be designed by optimizing the chemistry and density of interfacial catalytic fixed groups. If the electric field is dominant, the key design variables become the abruptness of the CEL/AEL junction and the thickness of the depleted region, to ensure field uniformity and localization. In practice, many high-performance BPMs use heterogeneous interfacial catalysts, often based on metal oxides or hydroxides [21,33,34,35,36,37,38], to reduce the apparent voltage required for water dissociation. While such catalysts can strongly improve performance, they also introduce complexity, cost, and mechanistic ambiguity. This is particularly relevant for acid–alkaline electrolysis, where one of the motivations for using BPMs is to reduce reliance on expensive or critical catalytic materials in the overall device [17,39,40,41,42]. Adding additional catalytic materials inside the BPM junction may improve WD but can also increase costs and complicate membrane design. This point is also connected to the frequent use of the standard 0.83 V water-dissociation reference. While this value is useful as the thermodynamic voltage associated with generating unit-activity acids and bases under an ideal ΔpH = 14 [2,16], it should not be interpreted as a fixed kinetic threshold for WD in a BPM junction. In neutral salt solutions, or in configurations where the membrane pH gradient is significantly lower than 14, WD has been observed at membrane voltages well below this value [43,44,45,46]. Therefore, the magnitude of the voltage alone is insufficient to describe WD enhancement; its spatial localization into a nanometric depleted junction, where an intense electric field is established, is the physically relevant factor [47].

In our previous work [48], we proposed a field-driven power-dissipation model that attributes WD enhancement to the intense electric field established in the depleted BPM junction, but reinterprets the role of this field differently from the Onsager theory of the SWE. The model is based on the idea that field-enhanced WD is an ion-mediated process through minority ions: $H_{3}O^{+}$ and $OH^{-}$ naturally present in liquid water at a concentration of $c_{ions}\;=\;10^{-7}\;mol\cdot L^{-1}$ due to autoprotolysis, rather than as a direct effect on water molecules. When an intense electric field is established in the depleted BPM junction, these minority ions undergo drift and continuously dissipate field-supplied power through viscous friction. The dissipated power per ion scale as:

(1) $$P\;=\;q\mu E^{2},$$

where $q\;=\;1.6\;\times \;10^{-19}\;C$ is the elementary charge, μ is the ionic mobility in m^2^·V^−1^·s^−1^, and E is the local electric field in $V\;m^{-1}$. This field-supplied dissipative power is not interpreted as bulk Joule heating, but as local entropy production at the molecular scale. Since WD is an endothermic chemical process that draws energy from its local environment, with a molecular dissociation energy $E_{d}\;=\;1.33\;\times \;10^{-19}\;J(\approx 0.83\;eV)$, we proposed that this dissipated power provides a local energetic pathway for increasing the probability of new water dissociation events [48]. The resulting rate expression predicts a quadratic dependence of the field-enhanced water dissociation rate constant $k_{d}(E)$ on the local electric field:

(2) $$k_{d}\left(E\right)=\left(\frac{c_{ions}}{c_{H2O,l}}\right)\times \frac{q\;\times \;\left({µ}_{{H3O}^{+}}\;+\;{µ}_{{OH}^{-}}\right)}{E_{d}}\;\times \;E^{2},$$

where

$k_{d}(E)$: Dissociation rate constant of water molecules as a function of the field E in (s^−1^);

c_ions_: Concentration of H_3_O^+^ and OH^−^ ions in water (10^−7^ mol·L^−1^ at 25 °C);

$\;c_{H2O,l}$: Concentration of free liquid water (55.5 mol·L^−1^ at 25 °C);

q: Absolute electric charge of H_3_O^+^ and OH^−^ ions (q = 1.6 × 10^−19^ C);

$\;\mu_{H3O+}$: Ionic mobility of the hydronium ion H_3_O^+^ (3.62 × 10^−7^ m^2^·V^−1^·s^−1^ at 25 °C);

$\;\mu_{OH-}$: Ionic mobility of the hydroxide ion OH^−^ (2.05 × 10^−7^ m^2^·V^−1^·s^−1^ at 25 °C);

E: The electric field in the BPM junction in (V·m^−1^);

$\;E_{d}$: Threshold dissociation energy per water molecule in (J), ($E_{d}$ = 1.33 × 10^−19^ J at 25 °C).

At 25 °C, Equation (2) reduces to:

(3) $$k_{d}(E)\;=\;1.23\;\times \;10^{-15}E^{2},$$

with $k_{d}(E)$ in $s^{-1}$ and E in $V\;m^{-1}$. This expression was shown to capture the order of magnitude required for $k_{d}$ under BPM-relevant fields using only measurable physical quantities at a given temperature [48]. Using a mass-balance equation [6,49], the WD current density $J_{WD}$ in the BPM junction with a thickness δ is given by:

(4) $$J_{WD}\;=\;F\;\times \;c_{H2O,j}\times k_{d}(E)\;\times \;\delta ,$$

where $J_{WD}$, F, $k_{d}(E)$, $c_{H2O,j}$, and δ are the WD current density in A·m^−2^, the Faraday constant 96,485 C·mol^−1^, the field-enhanced water dissociation rate constant in s^−1^, the water concentration in the junction in mol·m^−3^, and the thickness of the BPM junction in m, respectively. By relating the local field to the effective voltage drop across the active junction, $E\;\approx \;U_{j}/\delta$. Replacing $k_{d}(E)$ in Equation (4) by its Equation (2) expression leads to the quadratic junction law

(5) $$J_{WD}\;=\;KU_{j}^{2},$$

where $U_{j}$ is the effective voltage drop localized in the water-dissociation region in volts V, often referred to as WD overpotential ($\eta_{WD})$ in the recent literature [2,16]. The prefactor K (in mA cm^−2^ V^−2^) depends primarily on the effective water availability in the junction and the effective thickness over which the field is localized, and is given by [48]:

(6) $$K=\frac{F\;\times \;f\;\times \;q\;\times \;c_{ions}\;\times \;\left({µ}_{{H3O}^{+}}\;+\;{µ}_{{OH}^{-}}\right)}{\delta \;\times \;E_{d}},$$

where $f\;=\;c_{H_{2}O,j}/c_{H_{2}O,l}$ represents the effective water availability or hydration factor in the junction, and the other parameters are defined above. At 25 °C, when f = 1 and δ = 1 nm, the value of K is about 660 mA cm^−2^ V^−2^.

The initial validation of this model was limited to a two-electrode configuration experiment on a single commercial BPM and over a restricted current-density range. The present work extends that validation by investigating three commercial BPMs under two electrolyte configurations and relatively higher current densities. Four-electrode polarization measurements are performed under both acid/base and neutral salt conditions. Since real BPM polarization curves also contain contributions from co-ion and counter-ion crossover, internal concentration polarization, catalytic activity, and water supply limitations [22,45,50,51,52,53,54,55], a meaningful validation of the quadratic model must distinguish the WD-dominated regime from the transition regions that precede or follow it. The objectives are to determine whether the WD-dominated portions of the polarization curves follow the predicted $J_{WD}$ quadratic dependence, to compare the extracted K values across different membranes, and to clarify how membrane-specific junction properties affect the transition toward field-dominated WD. In addition, the model is extended to high current density by incorporating finite water transport to the junction through the AEL and CEL. This extension allows deviations from the ideal quadratic law at high current to be interpreted as the onset of water-supply limitation, linking the field-driven WD mechanism to practical high-current BPM operation.

The remainder of this article is organized as follows. Section 2 presents the experimental methods and the data-analysis framework and then introduces the analytical extension of the model to high-current water-transport limitations. Section 3 presents the experimental results followed by the interpretation of high-current deviations using the saturation model. Section 4 summarizes the main conclusions.

## 2. Materials and Methods

### 2.1. Experimental Methods

#### 2.1.1. Membranes

Three commercial bipolar membranes were investigated in this work and are denoted as BPM-1, BPM-2, and BPM-3 throughout the manuscript. BPM-1 corresponds to a bipolar membrane acquired from Alfa Chemistry (Holbrook, NY, USA), BPM-2 to a Fumasep FBM (FUMATECH BWT GmbH, Bietigheim-Bissingen, Germany) acquired from FuelCell Store (Bryan, TX, USA), and BPM-3 to a Neosepta-BPU acquired from ASTOM Corporation (Minato-ku, Tokyo, Japan). The membranes were selected to compare different commercial BPM architectures and interfacial behaviors under identical electrochemical conditions. BPM-1 and BPM-3 were used as received. BPM-2 was stored for several weeks in deionized water (DI) prior to testing, with regular water renewal to avoid excessive drying and to preserve membrane hydration. The main properties of the three BPMs are summarized in Table 1. These properties were compiled from supplier information when available and complemented with values reported in the literature.

#### 2.1.2. Four-Electrode H-Cell Configuration and Components

**Figure 2:**
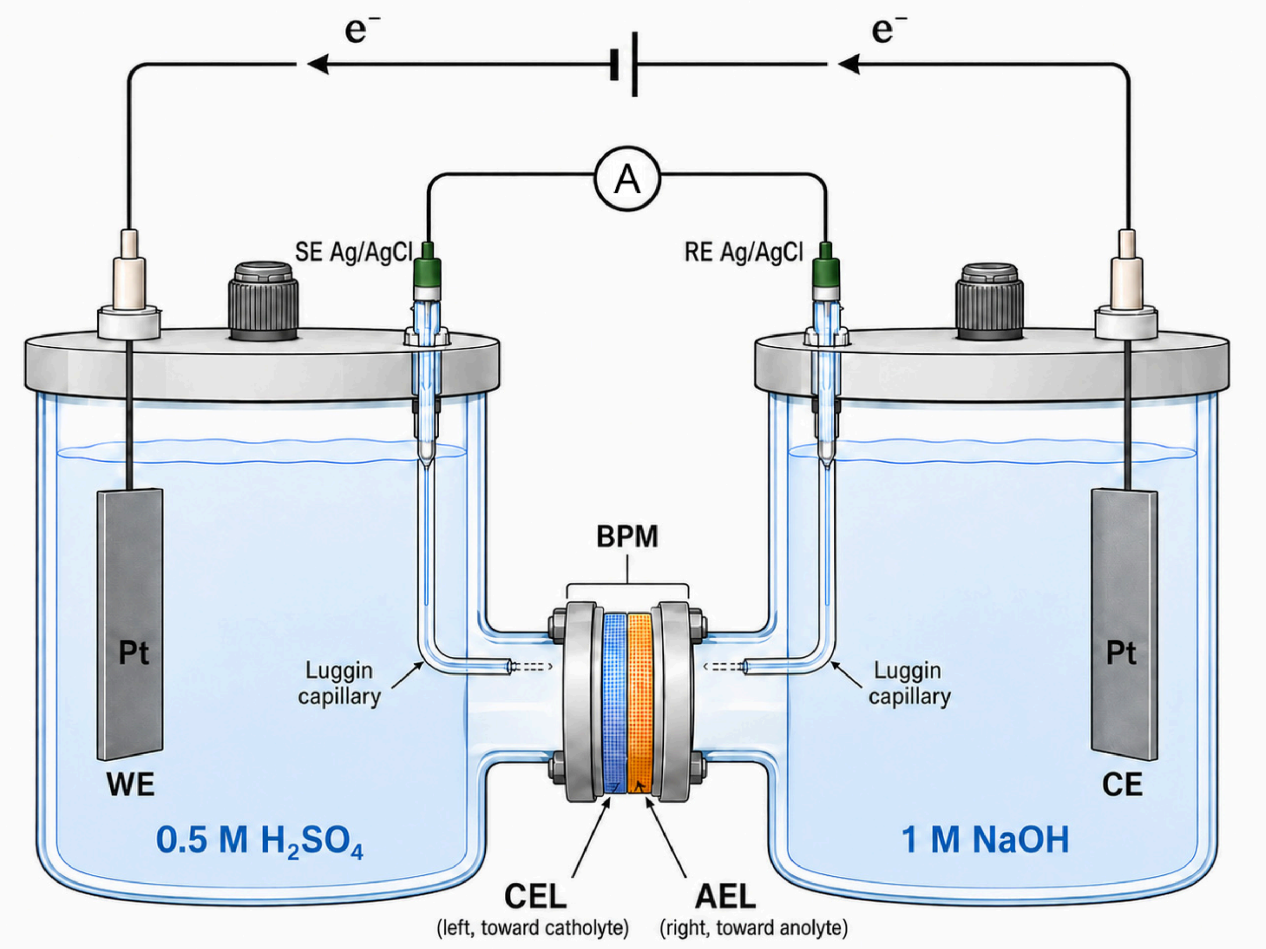
Four-electrode H-cell configuration used for BPM polarization measurements.

A schematic representation of the four-electrode H-cell configuration is shown in Figure 2. BPM samples were cut into approximately $1\;cm^{2}$ pieces and mounted between the two compartments of a glass H-cell (StonyLab, Nesconset, NY, USA). Each compartment had a volume of 20 mL. The membranes were clamped between PTFE gaskets, which defined a circular exposed membrane area of $0.79\;cm^{2}$. The CEL was oriented toward the catholyte side and the AEL toward the anolyte side for all experiments, corresponding to reverse-bias operation of the BPM. Platinum electrodes (StonyLab, Nesconset, NY, USA) with a geometric area of $1\;cm^{2}$ were used as the current-carrying electrodes. The working electrode (WE) was placed in the catholyte compartment, while the counter electrode (CE) was placed in the anolyte compartment. Before each experiment, the Pt electrodes were cleaned sequentially with acetone, deionized water, and alcohol, and then rinsed thoroughly with deionized water before use.

Two saturated Ag/AgCl reference electrodes (StonyLab, Nesconset, NY, USA) were used to measure the potential difference across the BPM. Before each measurement, the reference electrodes were immersed for at least 30 min in saturated KCl solution. The reference electrodes were positioned close to the BPM surfaces through Luggin capillaries in order to minimize the uncompensated electrolyte resistance included in the measured BPM voltage. The reference electrode on the catholyte side was used as the sense electrode (SE), while the reference electrode on the anolyte side was used as the reference electrode (RE), following the notation used in the schematic representation of the cell. All measurements were conducted at 25 °C.

For acid/base measurements, the catholyte contained $0.5\;M\;H_{2}SO_{4}$, and the anolyte contained 1.0 M NaOH. For neutral pH measurements, both compartments contained $0.5\;M\;Na_{2}SO_{4}$. All electrolytes were prepared using analytical-grade reagents and deionized water. The solutions were used under static conditions without recirculation during the linear sweep voltammetry (LSV) measurements. The measured pH difference in the acid/base configuration was slightly lower than the ideal value of 14, and typically ranged between approximately 13.56 and 13.84.

#### 2.1.3. Electrochemical Instrumentation and Four-Electrode Measurements

LSV measurements were performed using a VersaSTAT 4A potentiostat (AMETEK, Oak Ridge, TN, USA) in four-electrode configuration. The potential was swept at a scan rate of $5\;mV\;s^{-1}$. The measured BPM voltage corresponds to the potential difference between the two Ag/AgCl reference electrodes positioned close to the membrane surfaces. The LSV curves were automatically $iR_{s}$-corrected using the built-in VersaSTAT 4 compensation procedure. The current was normalized by the exposed membrane area of $0.79\;cm^{2}$, and the current density is reported in $mA\;cm^{-2}$. The use of a four-electrode configuration was essential to separate the BPM voltage from the electrode overpotentials associated with the hydrogen and oxygen evolution reactions [2,16]. The potential difference between the two reference electrodes was taken as the BPM voltage, $U_{BPM}$. For the acid/base experiments, the equilibrium BPM voltage was associated with the imposed pH gradient across the membrane. In an ideal BPM, this equilibrium voltage can be estimated from the Nernst relation

(7) $$U_{eq}\;\approx \;0.059\;\Delta pH$$

at room temperature. Experimentally, $U_{eq}$ was taken as the open-circuit or near-zero-current BPM voltage, $U_{oc}$, measured before the onset of significant reverse-bias current. The effective junction voltage $U_{j}$ used in the acid/base analysis was then defined as:

(8) $$U_{j}=U_{BPM}-U_{oc}.$$

This definition was applied to the acid/base experiments, where the macroscopic BPM equilibrium voltage is well defined by the pH gradient. In the neutral $Na_{2}SO_{4}$ experiments, ΔpH = 0 and the macroscopic equilibrium BPM voltage is close to zero; therefore, the neutral pH data were analyzed using an effective transition voltage $U_{wd}$, as described in the fitting procedure below. For clarity, reverse-bias voltages and current densities are reported as positive values in the polarization curves and fitting analysis.

For each BPM, several LSV curves were collected over current-density ranges up to $100–300\;mA\;cm^{-2}$, corresponding to the range where the WD-dominated response could be reliably identified. Representative curves were selected based on curve stability, reproducibility, absence of obvious artifacts, and suitability for the corresponding analysis. For the main acid/base validation, the final selected curves were used to compare the three BPMs under identical pH gradient conditions. For the high-current extension, additional representative curves that reached a higher current density were selected for BPM-1 and BPM-2. Error bars were included in the overlay figures as relative current-density uncertainties. The values used were based on the dispersion observed in the LSV datasets and applied consistently to the corresponding BPMs.

#### 2.1.4. Quadratic Fitting of the WD-Dominated Regime

The power-dissipation model predicts that, in the WD-dominated regime, the current density follows Equation (5). As discussed above in Section 1, the low-current region of real BPM polarization curves may include different contributions: co-ion and counter-ion crossover, internal concentration polarization, and early non-dominant WD. Therefore, a transition current $J_{t}$ was introduced to separate the mixed-current region from the WD-dominated region. This type of baseline or transition-current correction is consistent with the way BPM current–voltage curves are commonly interpreted, where the water-dissociation branch is distinguished from the preceding limiting-current or salt-transport contribution [50]. For the acid/base experiments, the fitting equation was:

(9) $$J-J_{t}\;=\;KU_{j}^{2},$$

or, using Equation (8):

(10) $$J-J_{t}\;=\;K(U_{BPM}-U_{oc}{)}^{2}$$

for $U_{BPM}\;>\;U_{BPM}\left(J_{t}\right)$. The current $J_{t}$ is not interpreted as the absolute onset of water dissociation. Rather, it is an operational transition current above which the water-dissociation contribution becomes dominant enough for the quadratic scaling to describe the measured current.

For neutral pH experiments, the macroscopic open-circuit BPM voltage is close to zero, and the equilibrium acid/base voltage reference used in the acid/base experiments is not applicable. Therefore, neutral pH data were analyzed using an effective transition voltage $U_{wd}$, defined as the potential above which the current–voltage response becomes well described by the quadratic form. The fitting equation was:

(11) $$\begin{array}{cccc} & J-J(U_{wd})\;=\;K(U_{BPM}-U_{wd}{)}^{2}. & & \end{array}$$

Here, $U_{wd}$ is treated as an empirical transition voltage where WD become dominant, not as a thermodynamic onset potential for WD. The fitting parameters K and $J_{t}$ were obtained by least-squares regression. The goodness of fit was evaluated using the coefficient of determination:

(12) $$R^{2}=1-\frac{\sum_{i}(J_{i}-J_{i,fit}{)}^{2}}{\sum_{i}(J_{i}-J_{i}{)}^{2}},$$

where $J_{i}$ is the experimental current density, $J_{i,fit}$ is the fitted current density, and $J_{i}$ is the mean current density over the fitted region.

### 2.2. Theoretical: Model Extension to High Current Density and Water-Transport Limitation

The quadratic field-dissipation model describes the intrinsic WD response of a hydrated BPM junction when sufficient water is locally available, corresponding to low current densities. This aspect was developed in our previous work [48]. At high current density, however, the field-driven WD rate may become faster than the rate at which water can be supplied to the junction through the AEL and CEL [22,54]. In this situation, the local water concentration in the junction can no longer be treated as constant, and the ideal quadratic law must be coupled to a water-transport balance. Water-transport limitations in BPMs have previously been discussed in the context of high-current operation, membrane dehydration, and upper limiting current behavior [52,54,55]. In the present work, this effect is incorporated directly into the quadratic field-dissipation model through a simple steady-state Fickian water-supply balance. The extension is based on the following assumptions:

1. Water reaches the BPM junction by diffusion through both the AEL and CEL.
2. Convective water transport is neglected.
3. A one-dimensional steady-state water concentration profile is assumed in each layer.
4. The water concentration decreases linearly from the outer membrane interfaces toward the junction.
5. Each layer is assumed, as a simplifying approximation, to supply half of the water concentration required at the junction.
6. The effective water concentration at the outer side of each layer is expressed through a water fraction $f_{w,i}$, where i=AEL,CEL.
7. The junction water concentration is written as:

(13) $$\begin{array}{cc} & c_{H_{2}O,j}\;=\;f\;c_{H_{2}O,l}\;, \end{array}$$

where $c_{H_{2}O,l}$ is the concentration of liquid water.

**Figure 3:**
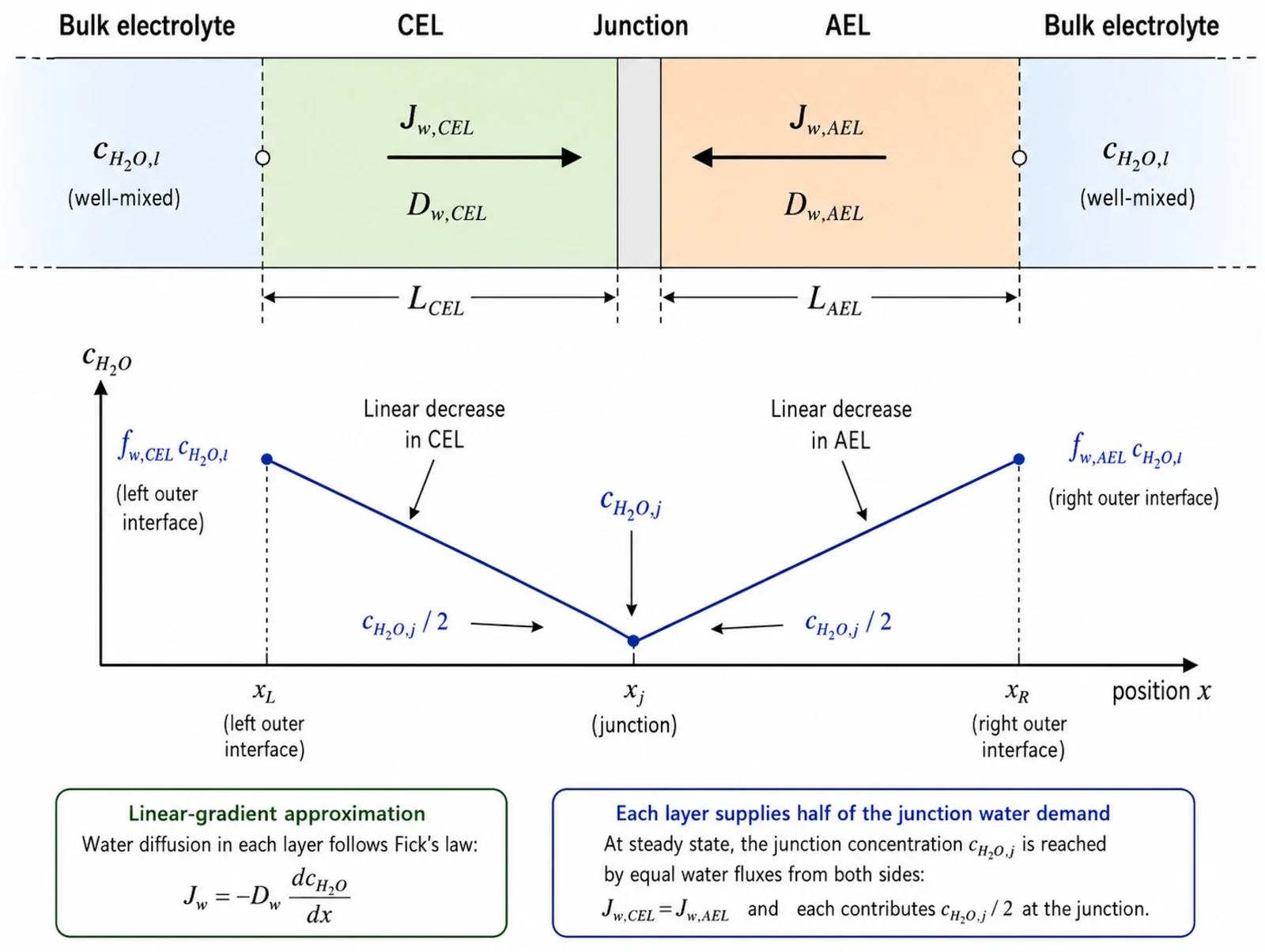
Schematic representation of the water-transport limitation in the BPM junction. The model assumes one-dimensional Fickian diffusion from both membrane layers toward the junction, and each layer is assumed to provide half of the water concentration required at the junction.

The physical schematic is illustrated in Figure 3. The water concentration at the outer interface of layer i is:

(14) $$\begin{array}{cc} & c_{H_{2}O,i}^{outer}\;=\;f_{w,i}c_{H_{2}O,l} \end{array}.$$

Using a linear-gradient approximation, the diffusive water flux through each layer is given by Fick’s law:

(15) $$J_{w,i}=\frac{D_{w,i}}{L_{i}}\left(f_{w,i}c_{H_{2}O,l}\frac{c_{H_{2}O,j}}{2}\right),$$

where $D_{w,i}$ is the water diffusion coefficient in layer i, and $L_{i}$ is the layer thickness. The term $c_{H_{2}O,j}/2$ reflects the simplifying assumption that the water demand at the junction is supplied symmetrically from both sides. At steady state, the water concentration in the junction does not accumulate:

(16) $$\frac{dc_{H_{2}O,j}}{dt}\;=\;0.$$

Therefore, the total incoming water flux must balance the water consumption rate associated with WD:

(17) $$\begin{array}{cccc} & J_{w,AEL}+J_{w,CEL}=\frac{J_{WD}}{F} & & \end{array},$$

where $J_{WD}/F$ is the molar water consumption flux corresponding to the WD current density. Substituting Equation (15) into Equation (17), and using Equation (13), gives:

(18) $$\frac{J_{WD}}{F}=c_{H_{2}O,l}\left[\frac{D_{w,AEL}}{L_{AEL}}f_{w,AEL}+\frac{D_{w,CEL}}{L_{CEL}}f_{w,CEL}-\frac{f}{2}\left(\frac{D_{w,AEL}}{L_{AEL}}\frac{D_{w,CEL}}{L_{CEL}}\right)\right].$$

For compactness, we define:

(19) $$\begin{array}{cccc} & A=\frac{D_{w,AEL}}{L_{AEL}}f_{w,AEL}+\frac{D_{w,CEL}}{L_{CEL}}f_{w,CEL} & & \end{array}$$

and

(20) $$\begin{array}{cc} & B=\frac{D_{w,AEL}}{L_{AEL}}\;+\;\frac{D_{w,CEL}}{L_{CEL}} \end{array}.$$

Equation (18) then becomes

(21) $$\frac{J_{WD}}{F}=c_{H_{2}O,l}\left(A\frac{f}{2}B\right).$$

The maximum water-supply-limited current density is reached when the effective junction water concentration tends to zero, i.e., f→0. This defines

(22) $$J_{lim,2}\;=\;Fc_{H_{2}O,l}\left[\frac{D_{w,AEL}}{L_{AEL}}f_{w,AEL}\frac{D_{w,CEL}}{L_{CEL}}f_{w,CEL}\right],$$

or, equivalently:

(23) $$J_{lim,2}\;=\;Fc_{H_{2}O,l}A.$$

The conductance-weighted equilibrium water fraction supplied by the two layers is defined as:

(24) $$f_{eq}=\frac{\left(\frac{D_{w,AEL}}{L_{AEL}}\right)f_{w,AEL}\;+\;\left(\frac{D_{w,CEL}}{L_{CEL}}\right)f_{w,CEL}}{\left(\frac{D_{w,AEL}}{L_{AEL}}\right)+\left(\frac{D_{w,CEL}}{L_{CEL}}\right)}=\frac{A}{B}.$$

Combining Equations (21)–(24) gives the current-dependent junction water fraction:

(25) $$f(J_{WD})\;=\;2f_{eq}\left(1\frac{J_{WD}}{J_{lim,2}}\right).$$

This expression requires:

(26) $$0\;\leq \;f(J_{WD}),$$

and therefore

(27) $$J_{WD}\;\leq \;J_{lim,2}.$$

When finite water supply is considered, the effective water fraction becomes current-dependent. Substituting Equation (25) into K of Equation (6) and then into Equation (5) of the quadratic WD law gives

(28) $$J_{WD}\;=\;J_{WD,0}\left(U_{j}\right)\left(1\frac{J_{WD}}{J_{lim,2}}\right)\;,$$

with

(29) $$J_{WD,0}\left(U_{j}\right)=2f_{eq}K_{WD}U_{j}^{2}.$$

is the intrinsic field-driven WD current in the absence of water-transport limitation, and $K_{WD}\;=\;K/f$ is the quadratic coefficient K of Equation (6), excluding the water-availability factor. For consistency with the quadratic fits used throughout this work, the effective prefactor $2f_{eq}K_{WD}$ is denoted simply as K, which represents the experimentally fitted WD prefactor that already includes the equilibrium water-availability factor $f_{eq}$. Thus, Equation (29) is written as

(30) $$J_{WD,0}\left(U_{j}\right)=KU_{j}^{2}.$$

Rearranging Equation (28) gives the saturation form:

(31) $$J_{WD}\left(U_{j}\right)=\frac{J_{lim,2}J_{WD,0}\left(U_{j}\right)}{J_{lim,2}\;+\;J_{WD,0}\left(U_{j}\right)}.$$

Equation (31) preserves the ideal quadratic behavior when

(32) $$J_{WD,0}(U_{j})\;\ll \;J_{lim,2},$$

so that

(33) $$J_{WD}(U_{j})\;\approx \;J_{WD,0}\left(U_{j}\right).$$

At high current density, when

(34) $$J_{WD,0}(U_{j})\;\gg \;J_{lim,2},$$

the current approaches

(35) $$J_{WD}(U_{j})\;\approx \;J_{lim,2},$$

which corresponds to a water-transport-limited regime.

The mathematical inflection point of the saturation curve is obtained when

(36) $$J_{WD,0}(U_{j})\;=\;\frac{1}{3}J_{lim,2}.$$

Substituting this $J_{WD,0}(U_{j})$ value into Equation (30) gives

(37) $$J_{WD,inf}\;=\;\frac{1}{4}J_{lim,2}.$$

Thus, $J_{WD,inf}\;=\;0.25\;J_{lim,2}$, and a deviation from the ideal quadratic law can appear well before the true limiting current is reached. This high-current extension is not intended to independently determine $D_{w,i}$, $L_{i}$, and $f_{w,i}$ from polarization data alone. Instead, it provides a physically constrained framework for interpreting deviations from the ideal quadratic law when water transport to the BPM junction becomes limiting. Compared with earlier treatments of water-transport limitations [22,54], the present formulation directly couples the field-driven WD law and the finite water-supply constraint in a single predictive expression, while keeping the physical meaning of each parameter explicit.

## 3. Results and Discussion

### 3.1. Acid/Base BPM Polarization Behavior and Quadratic Validation

**Figure 4:**
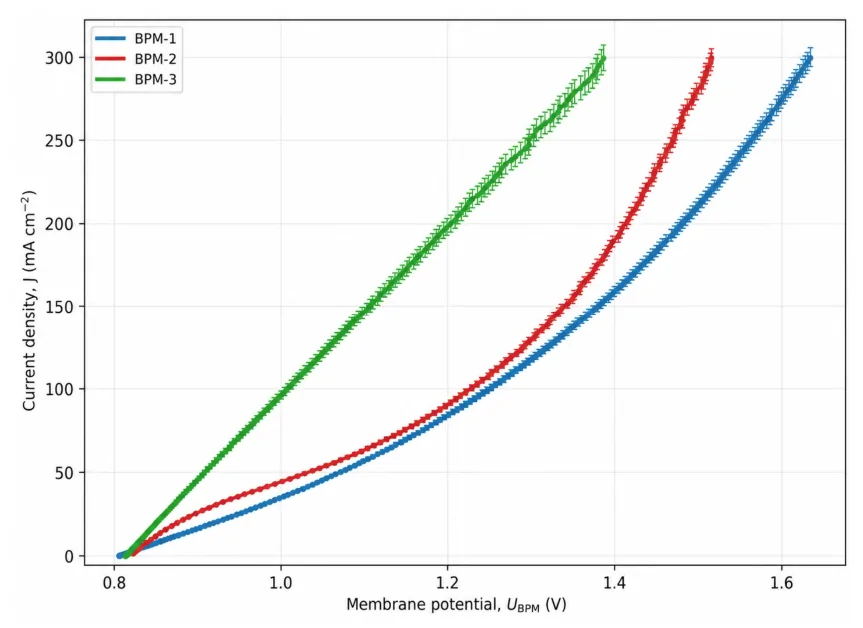
Acid/base polarization curves with $J\;vs.U_{BPM}$ of the selected BPMs up to 300 mA cm^−2^.

Figure 4 shows the final acid/base polarization curves of the three BPMs investigated in this work, up to $300\;mA\;cm^{-2}$. The curves are plotted with error bars as $J\;vs.\;U_{BPM}$. The overall shapes are consistent with BPM current–voltage measurements in acid–alkali systems, where the response is typically more direct than in neutral salt solutions and may show a reduced or absent low-current plateau depending on membrane structure and electrolyte concentration [60]. Nevertheless, the three BPMs exhibit markedly different polarization responses. BPM-1 shows a relatively sharp transition from a low-current region to a rapidly increasing current. BPM-2 displays similar behavior, although its current increase is shifted and slightly less ideal than BPM-1. In both cases, the curves exhibit a clear nonlinear rise after a short transition region, consistent with the development of a field-driven WD-dominated regime after junction depletion.

BPM-3 behaves differently. Its current increases more gradually and almost linearly over a broad current range. This behavior is consistent with the presence of an interfacial catalyst in this membrane [1,58,59]. As an indicative comparison, at $J=100\;mA\;cm^{-2}$, BPM-3 required the lowest membrane potential, followed by BPM-2 and BPM-1. In such a membrane, the low- and intermediate-current response may be governed not only by the electric-field-driven process considered in the present model, but also by catalytic proton-transfer pathways and heterogeneous interfacial effects.

The differences observed in Figure 4 show that a BPM polarization curve should not be interpreted as a single-mechanism response over its entire current range. At low current density, the measured current can involve several ionic species, and it is generally governed by counter-ion and co-ion crossover. Thus, the quadratic field-dissipation model is expected to describe the portion of the curve where WD becomes the dominant contribution to the measured current, rather than the whole polarization curve from the first measurable current.

**Figure 5:**
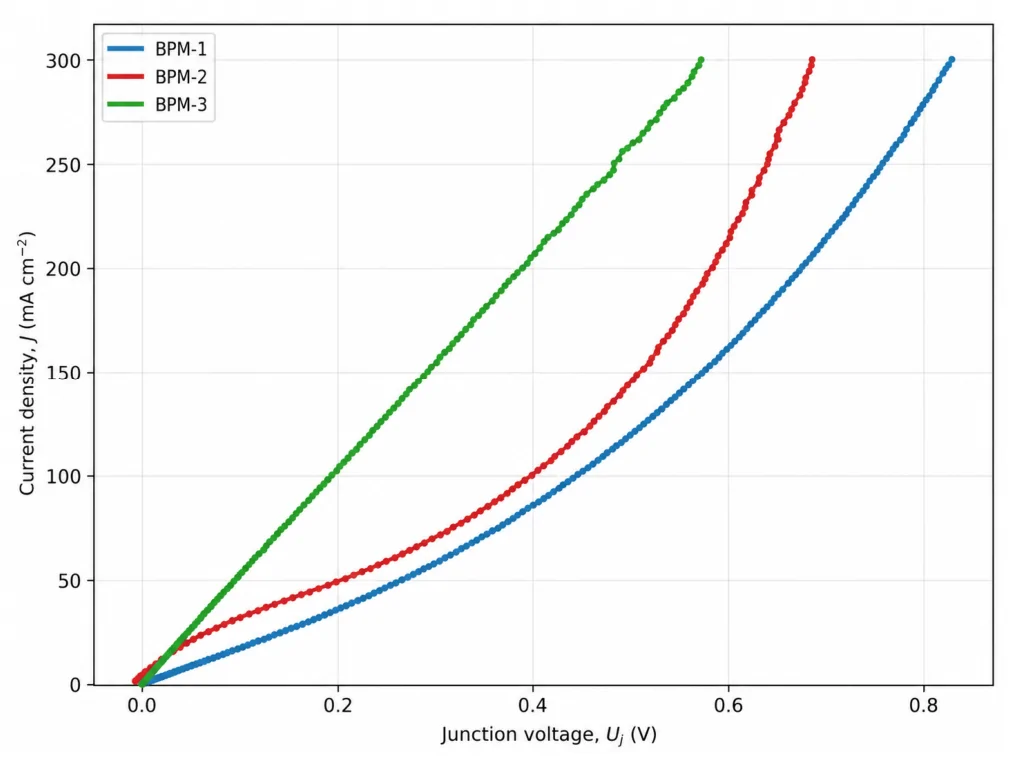
Acid/base polarization curves plotted as J versus $U_{j}$.

To compare the membranes on the basis of the driving voltage relevant to the junction response, the acid/base polarization curves were then replotted using Equation (8) as J versus $U_{j}$ (Figure 5). This representation removes the equilibrium BPM voltage associated with the imposed pH gradient and provides the voltage scale used for the quadratic analysis. The resulting J–$U_{j}$ curves were then fitted in the WD-dominated region using Equation (9) from Section 2.1.4, where $J_{t}$ is the transition current separating the mixed-current region from the WD-dominated region. The extracted fitting parameters for each are summarized in Table 2.

**Figure 6:**
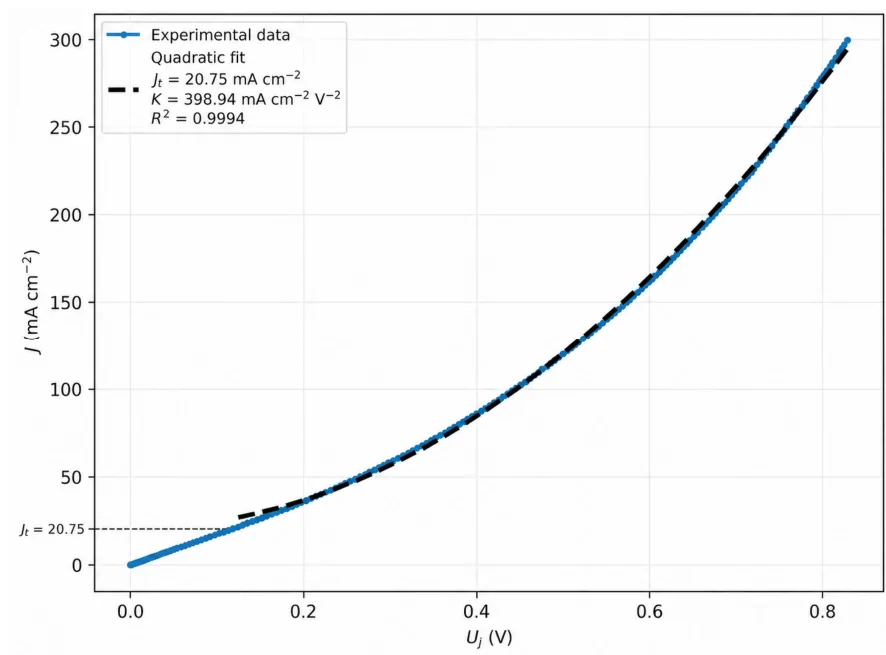
Quadratic fit of BPM-1 under acid/base conditions.

**Figure 7:**
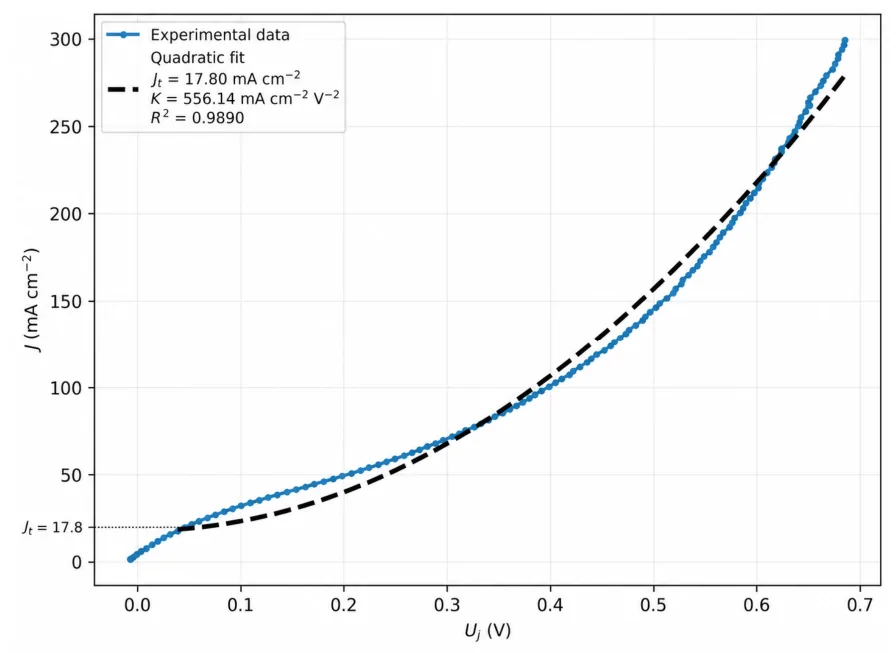
Quadratic fit of BPM-2 under acid/base conditions.

Figure 6 and Figure 7 show the quadratic fits obtained for BPM-1 and BPM-2, respectively. BPM-1 provides the clearest validation of the quadratic model, with $R^{2}=0.9994$ and $K\;=\;398.94\;mA\;cm^{-2}\;V^{-2}$. BPM-2 also follows the quadratic law, with $R^{2}\;=\;0.9890$ and a higher prefactor of $K\;=\;556.14\;mA\;cm^{-2}\;V^{-2}$. The transition currents are relatively low for both membranes$-J_{t}=20.75\;mA\;cm^{-2}$ for BPM-1 and $J_{t}\;=\;17.80\;mA\;cm^{-2}$ for BPM-2—but slightly higher than the typical limiting current densities observed in BPMs in neutral salt configurations. These $J_{t}$ values and the absence of a well-defined limiting current density may indicate a higher co-ion leakage due to concentrated solutions in this acid/base experiment [60,61], a higher co-ion and counter-ion crossover [52], or possible recombination of $H^{+}$ and $OH^{-}$ before complete depletion of the bipolar junction [50,52]. A possible WD contribution in this pre-transition region cannot be excluded, as reported in previous BPM studies [45,52]; however, fitting this low-current region with the present quadratic model, either alone or combined with a linear term, did not yield a physically meaningful quadratic contribution. Therefore, $J_{t}$ was retained as an operational boundary above which the WD contribution becomes dominant enough to be described by the quadratic law.

The difference between the fitted K values can be interpreted within the physical structure of the model. Since K scales with the effective water availability in the junction and inversely with the effective active thickness, a higher K may reflect either a more hydrated junction, a smaller junction thickness δ, or both. This is consistent with the available membrane properties from Table 1: BPM-1 has a reported water content in the range of 20–30%, whereas BPM-2 has a reported water content close to 32.5%. The higher K of BPM-2 may therefore be partly related to higher water availability, although a thinner effective junction cannot be excluded. The value obtained here for BPM-2 is also close to the prefactor previously reported for Fumasep FBM in our proof-of-concept study [48], $K\;\approx \;505\;mA\;cm^{-2}\;V^{-2}$. The slightly higher value obtained in the present work may arise from the improved four-electrode configuration, which measures the BPM voltage more directly, whereas the previous two-electrode configuration required reconstruction of the junction voltage by subtracting electrode overpotentials and ohmic contributions. Overall, the fitted K values for BPM-1 and BPM-2 remain within the expected range of the model and are physically interpretable in terms of hydration and active junction thickness. Together with the high $R^{2}$ values, these results provide strong experimental support for the quadratic field-dissipation law in non-catalytic BPM junctions.

**Figure 8:**
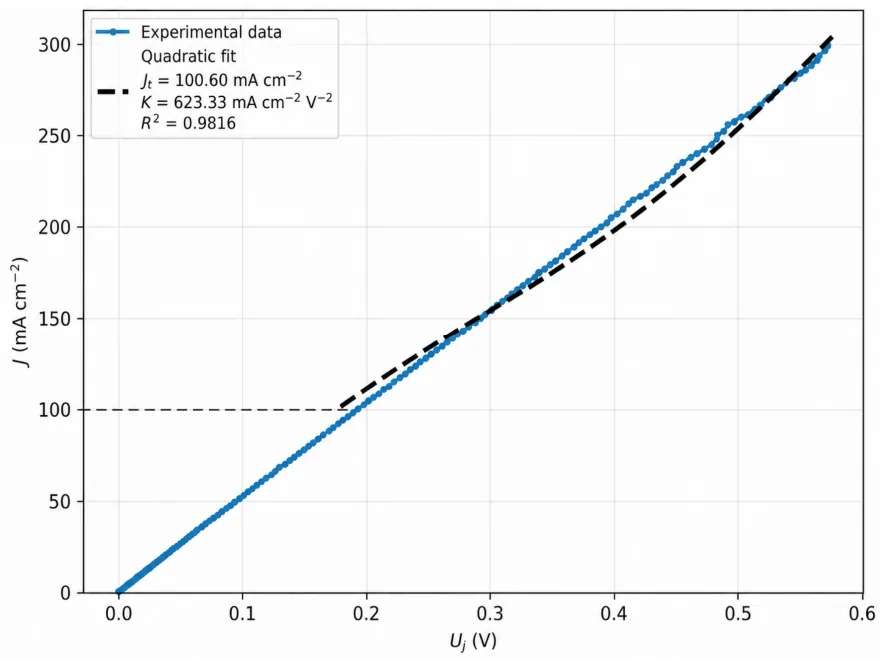
Quadratic fit of BPM-3 under acid/base conditions.

**Figure 9:**
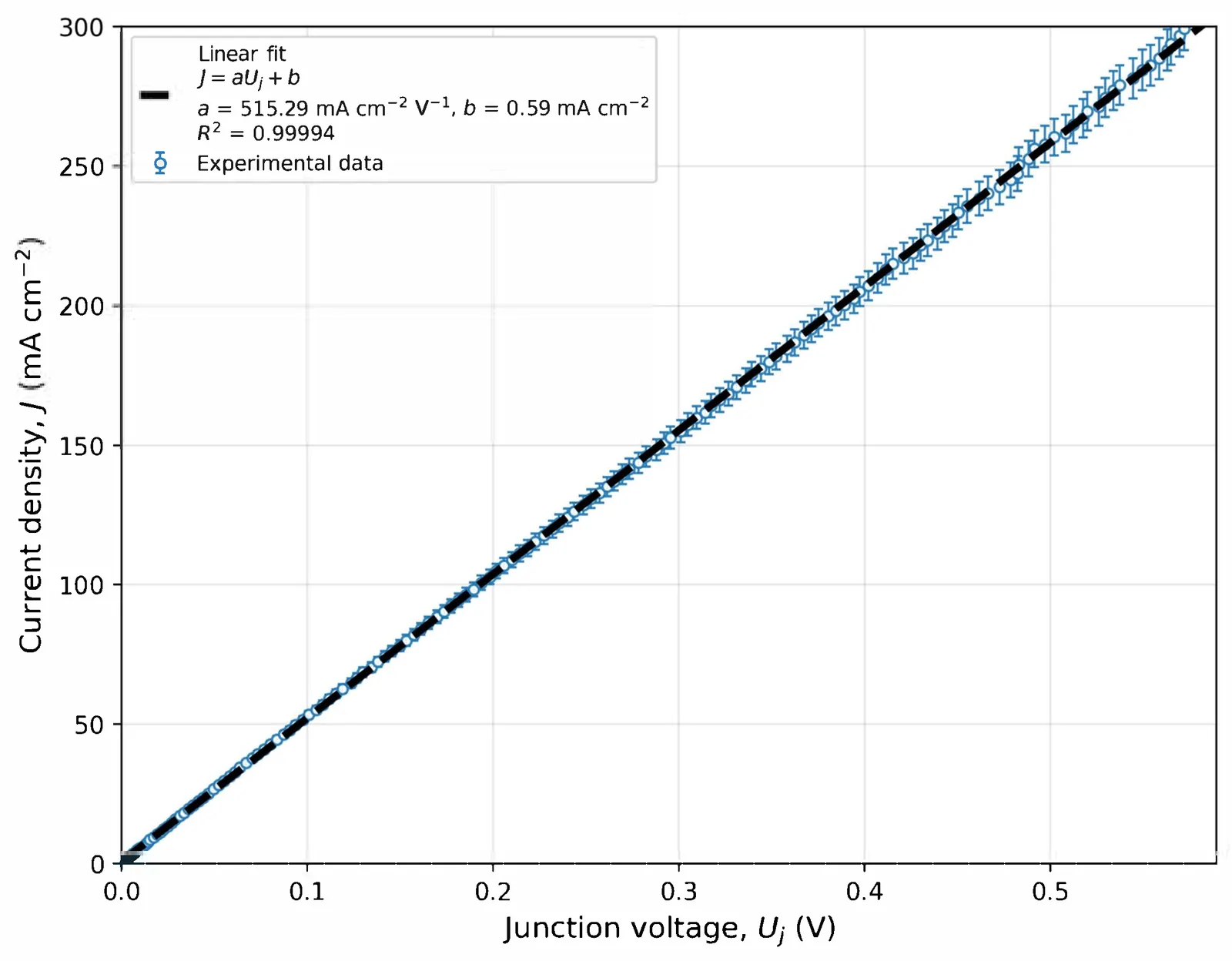
Linear fit of BPM-3 under acid/base conditions.

In contrast to BPM-1 and BPM-2, BPM-3 does not provide a straightforward validation of the quadratic field-dissipation law under acid/base conditions. Although a high-current quadratic fit (Figure 8) can be obtained for BPM-3, with $K\;=\;623.33\;mA\;cm^{-2}\;V^{-2}$ and $R^{2}\;=\;0.9816$, the overall polarization curve is more accurately described by a nearly linear response over the full investigated range, as shown in Figure 9. The linear fit gives $R^{2}\;=\;0.99994$, indicating that the dominant macroscopic response of BPM-3 under acid/base operation is not purely quadratic. This behavior is consistent with the known presence of a catalyst interfacial layer in Neosepta-type BPMs [1,58,59]. In such membranes, the measured current can reflect a complex balance between catalytic proton-transfer pathways, ion transport, and field-assisted WD. However, this balance is not trivial. Several studies have suggested that an interfacial catalyst can accelerate WD by providing proton-transfer sites, but if the catalytic layer is too thick or spatially extended, it may distribute the potential drop over a larger distance and weaken or screen the local electric field that would otherwise be concentrated in a sharp nanometric junction [62,63]. Conversely, thinner or some metal oxide catalysts may also locally concentrate the field over nanometric regions, creating possible field/catalyst synergy [33,36].

Within the present power-dissipation framework, a catalytic contribution could in principle be reflected by a lower effective dissociation energy $E_{d}$, which would increase the prefactor K. However, applying this interpretation quantitatively to BPM-3 would require independent knowledge of the catalyst thickness, morphology, and local field distribution inside the interfacial layer. Such information is not available in the present study. Consequently, the quadratic high-current fit of BPM-3 should be interpreted cautiously. It may suggest that a field-driven contribution becomes increasingly relevant at higher current density, but it cannot be considered as direct evidence of a purely field-dominated junction as in BPM-1 and BPM-2. Rather, BPM-3 illustrates a distinct case where catalytic and field-driven effects may coexist. In this sense, the quadratic model remains useful not only as a validation tool for abrupt, field-dominated BPM junctions, but also as a diagnostic framework: a clear dominant quadratic response suggests that the electric field controls the WD regime, whereas a weak, delayed, or masked quadratic signature may indicate that additional catalytic or structural mechanisms dominate the response.

### 3.2. Neutral Salt Validation of the Quadratic WD Response

**Figure 10:**
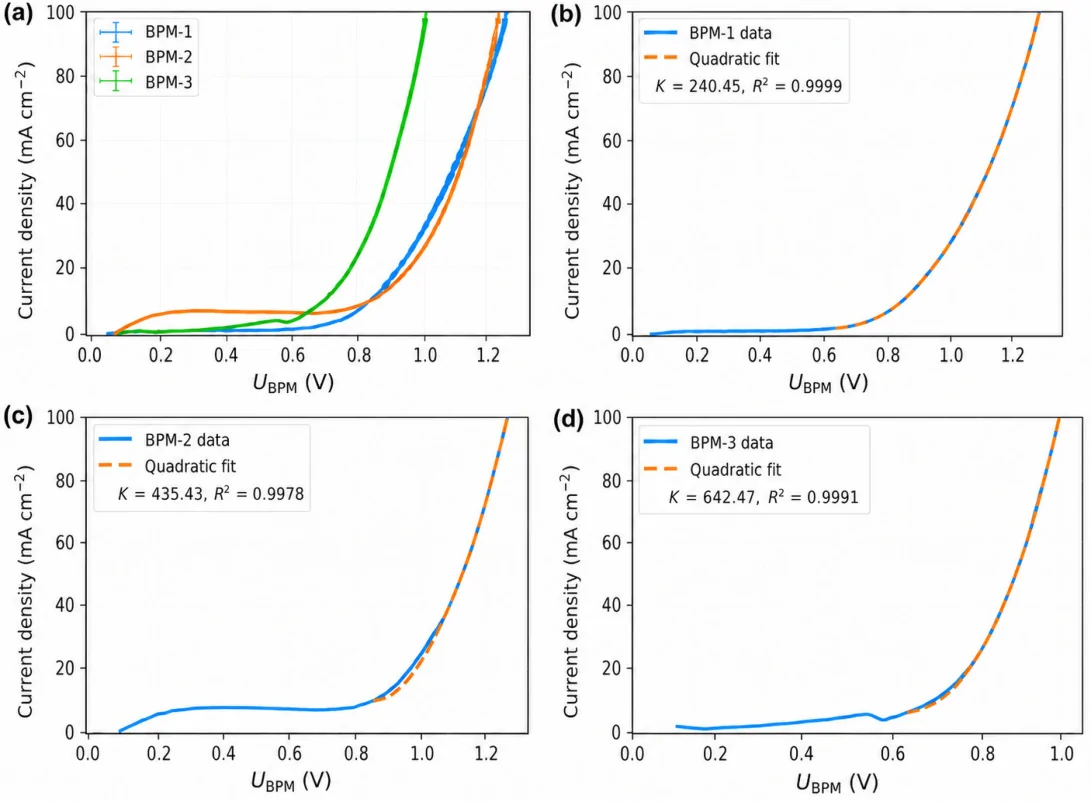
Polarization curves of BPM-1, BPM-2, and BPM-3 in $0.5\;M\;Na_{2}SO_{4}$: (**a**) the overlay of the three BPMs up to $100\;mA\;cm^{-2}$. Panels (**b**–**d**): quadratic fits of the WD-dominated region using Equation (11).

To further evaluate the quadratic field-dissipation law, the three BPMs were also tested in neutral $0.5\;M\;Na_{2}SO_{4}$. In this configuration, the macroscopic pH gradient across the BPM is absent, and the equilibrium BPM voltage is therefore close to zero. The polarization curves were analyzed using an effective transition voltage $U_{wd}$, as defined in Section 2, and the WD-dominated region was fitted using Equation (11). Figure 10a shows the polarization behavior of the three BPMs in neutral $0.5\;M\;Na_{2}SO_{4}$. Compared with the acid/base configuration, the neutral salt curves exhibit a markedly different shape. A broad limiting-current plateau is clearly observed before the rapid increase in current associated with water dissociation. This behavior contrasts with the acid/base experiments, where the low-current plateau was not clearly resolved and the transition current $J_{t}$ corresponded instead to an operational boundary between a mixed-current region and a WD-dominated regime. In neutral salt, the transition toward WD is therefore more clearly identified after the plateau, from an effective voltage $U_{wd}$, above which the current rises sharply.

The effective transition voltages extracted from the fits were $U_{wd}\;=\;0.63\;V$ for BPM-1, $U_{wd}\;=\;0.80\;V$ for BPM-2, and $U_{wd}\;=\;0.63\;V$ for BPM-3. These values are not interpreted as thermodynamic onset potentials, but as empirical voltages marking the beginning of the WD-dominated fitting region. The fact that the three BPMs exhibit a $U_{wd}\;<\;0.83\;V$ is consistent with several BPM studies reporting water dissociation or strong WD-related current increases below the ideal 0.83 V acid/base thermodynamic reference, especially under neutral or reduced pH gradient configurations [43,44,45,64]. This reinforces the idea that 0.83 V should be treated as a thermodynamic reference rather than as a universal kinetic threshold for WD. The onset of WD depends on the local field, junction depletion, hydration state, and membrane structure.

The fitted curves for each BPM are shown in Figure 10b–d, and the extracted parameters are summarized in Table 3. All the three BPMs show a very strong quadratic dependence in neutral salt conditions, once $U_{wd}$ is exceeded. BPM-1 gives $K\;=\;240.45\;mA\;cm^{-2}\;V^{-2}$ with $R^{2}\;=\;0.9999$. BPM-2 gives $K\;=\;435.43\;mA\;cm^{-2}\;V^{-2}$ with $R^{2}\;=\;0.9978$. BPM-3 gives $K\;=\;642.47\;mA\;cm^{-2}\;V^{-2}$ with $R^{2}\;=\;0.9991$. These excellent fits provide strong validation of the quadratic field-dissipation law in neutral electrolyte. Unlike the acid/base case, where BPM-3 displayed an almost linear global response, the neutral salt configuration reveals a clear quadratic WD-dominated branch for all three membranes. This suggests for BPM-3 that a catalyst-containing BPM can still display an apparent quadratic branch under specific electrolyte conditions, while not behaving as a purely field-dominated junction in acid/base operation.

The lower K values obtained in neutral $Na_{2}SO_{4}$ for BPM-1 and BPM-2 can be rationalized from the structure of the model, where K depends on the effective water availability in the junction and on the effective junction thickness. For BPM-1, K decreases by approximately 40% from acid/base to neutral salt, while for BPM-2, the decrease is approximately 22%. A first possible explanation is lower effective hydration in the neutral salt configuration, since the salt-ion-exchanged form of the AEL or CEL takes up less water than the corresponding hydronium or hydroxide form [52,65]. This directly supports the possibility that the effective water availability in the junction would be lower in $Na_{2}SO_{4}$ than in the acid/base configuration. A second possibility is a larger effective depleted region in neutral salt. In the acid/base configuration, the CEL and AEL are already supplied with highly mobile $H^{+}$ and $OH^{-}$, respectively, and these ions can more efficiently compensate the fixed charges near the junction. This may suggest a narrower depleted or space charge region than in neutral salt. Since K decreases when the active junction thickness δ increases, a broader depletion region in neutral salt would also contribute to the lower K values. A third contribution may arise from the transport mode of the WD products outside the junction, which may lead to a concentration polarization [45,52]. In acid/base operation, the adjacent phases already contain high concentrations of $H^{+}$ and $OH^{-}$, so diffusion of newly generated WD products away from the junction is not expected to be the main driving force. In neutral salt, however, $H^{+}$ and $OH^{-}$ are generated locally at the BPM junction into an environment where their bulk concentrations are low; this creates concentration gradients that can make diffusion contribute significantly to their transport outside the junction. Bui et al. [52] reported that, at relatively low current density, for ion-exchange layers in contact with neutral electrolyte, most of the hydronium or hydroxide current can be diffusion-driven, whereas in strongly acidic or alkaline environments, their transport is dominated by migration. Since the present $KU_{j}^{2}$ term represents the field-driven contribution, a stronger diffusive contribution outside the junction in neutral salt can lower the apparent K extracted from the macroscopic $J-U_{BPM}$ fit.

BPM-3 differs from BPM-1 and BPM-2 because its K value is nearly unchanged between neutral salt and acid/base conditions, with only an approximately 3% difference. This may indicate that the catalyst-containing interfacial region imposes a local WD environment that is less sensitive to the external electrolyte configuration. However, because BPM-3 shows a predominantly linear acid/base response, this interpretation remains qualitative. The result mainly shows that a quadratic branch can still appear in neutral salt for a catalyst-containing BPM, while the dominant macroscopic response in acid/base operation may be governed by catalyst-mediated or heterogeneous interfacial transport.

Overall, the neutral $Na_{2}SO_{4}$ experiments strongly support the quadratic field-dissipation model. The three BPMs display excellent parabolic fits after $U_{wd}$, with realistic prefactors and $R^{2}$ values close to unity. At the same time, the differences between the fitted K values in acid/base and neutral salt conditions show that the BPM water-dissociation response depends not only on membrane identity, but also on the external electrolyte conditions and their associated pH boundary conditions.

### 3.3. High-Current Deviation and Water-Transport-Limited Extension

**Figure 11:**
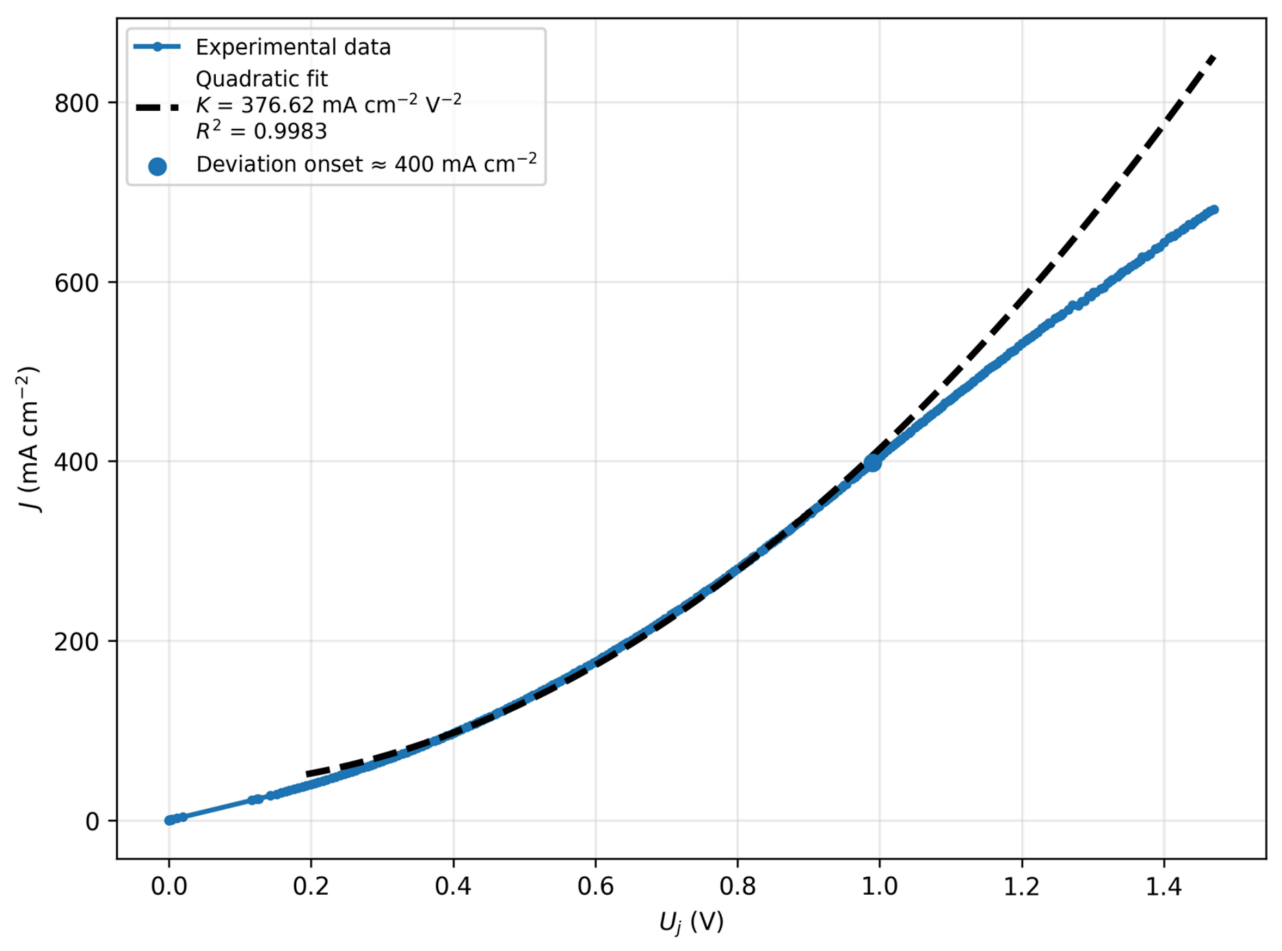
High-current deviation from the quadratic continuation for BPM-1.

**Figure 12:**
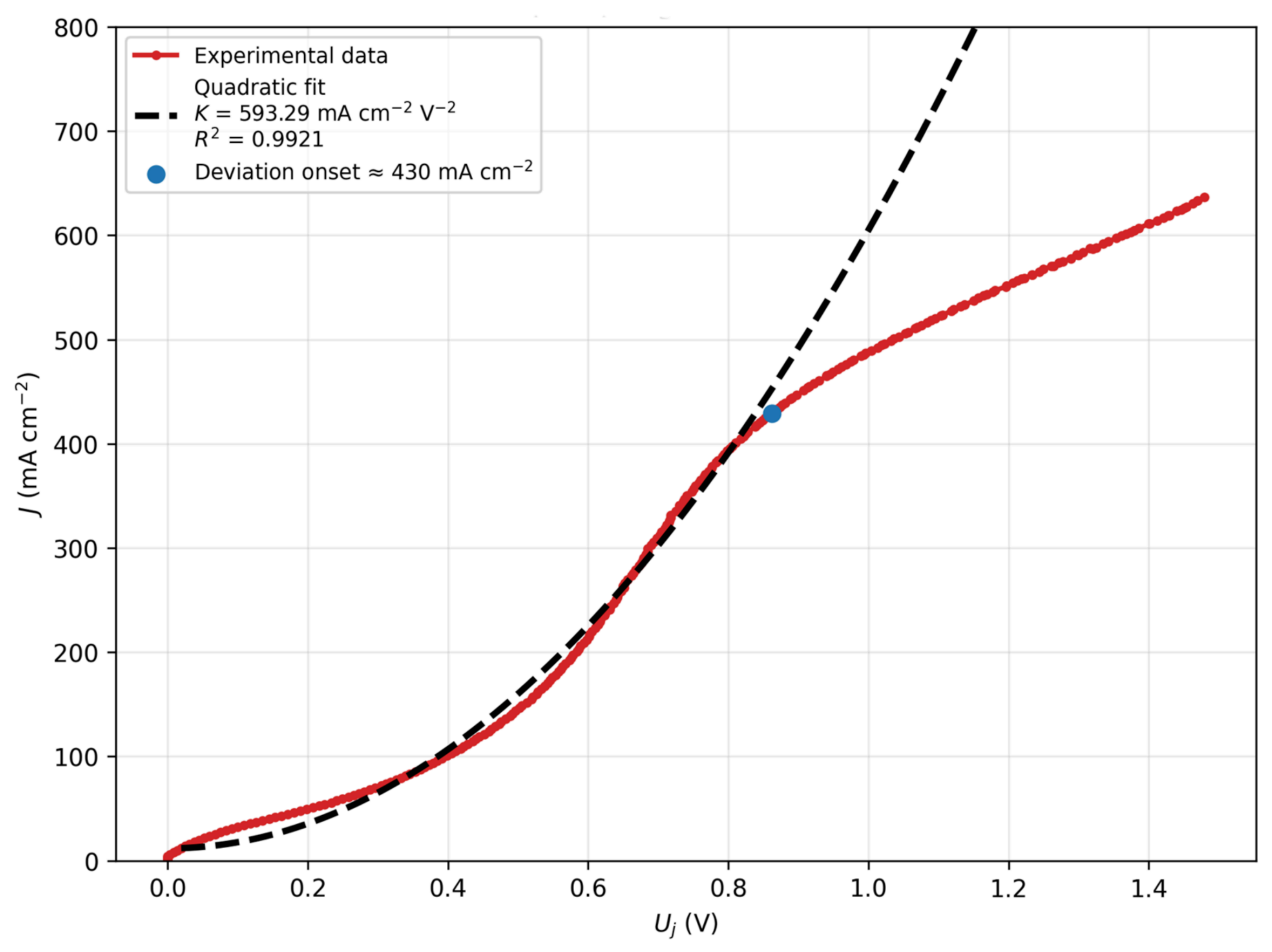
High-current deviation from the quadratic continuation for BPM-2.

After validating the quadratic WD law in the WD-dominated regime, BPM-1 and BPM-2 were further analyzed in an acid/base setup at higher current density to evaluate whether the deviation from the ideal quadratic response could be described by the water-transport-limited extension introduced in Section 2.2. BPM-3 was not used for this part of the analysis because its acid/base response was dominated by a nearly linear behavior and therefore does not provide a clean field-dominated reference for the high-current extension. Figure 11 and Figure 12 show the high-current behavior of BPM-1 and BPM-2, respectively. In both cases, the current initially follows the extrapolated quadratic law, confirming that the same field-driven WD mechanism remains active beyond the current range used for the main validation. At higher current density, however, the experimental curves progressively bend below the ideal quadratic prediction. This deviation is consistent with the physical picture of the extended model: as $U_{j}$ increases, the intrinsic field-driven WD rate becomes faster than the rate at which water can be supplied to the depleted junction through the CEL and AEL. The junction water concentration then decreases, and the apparent current approaches a water-supply-limited response rather than continuing to increase indefinitely as $KU_{j}^{2}$.

For BPM-1, the high-current quadratic fit gives $K\;=\;376.62\;mA\;cm^{-2}\;V^{-2}$, close to the value obtained in the main acid/base validation. The onset of deviation from the ideal quadratic branch occurs at approximately: $J_{WD,inf}\;\approx \;400\;mA\;cm^{-2}$. Using Equation (37) derived in Section 2.2 leads to $J_{lim,2}\;\approx \;1600\;mA\;cm^{-2}$. For BPM-2, the high-current fit gives $K\;=\;593.29\;mA\;cm^{-2}\;V^{-2}$, also close to the value extracted from the acid/base WD-dominated region. The deviation from the quadratic branch appears slightly later, at approximately $J_{WD,inf}\;\approx \;430\;mA\;cm^{-2}$, which gives $J_{lim,2}\;\approx \;1720\;mA\;cm^{-2}$. The slightly higher $J_{lim,2}$ of BPM-2 is physically reasonable considering its higher water content (Table 1) and its higher fitted K, both of which suggest more favorable water availability and/or a sharper active junction.

**Figure 13:**
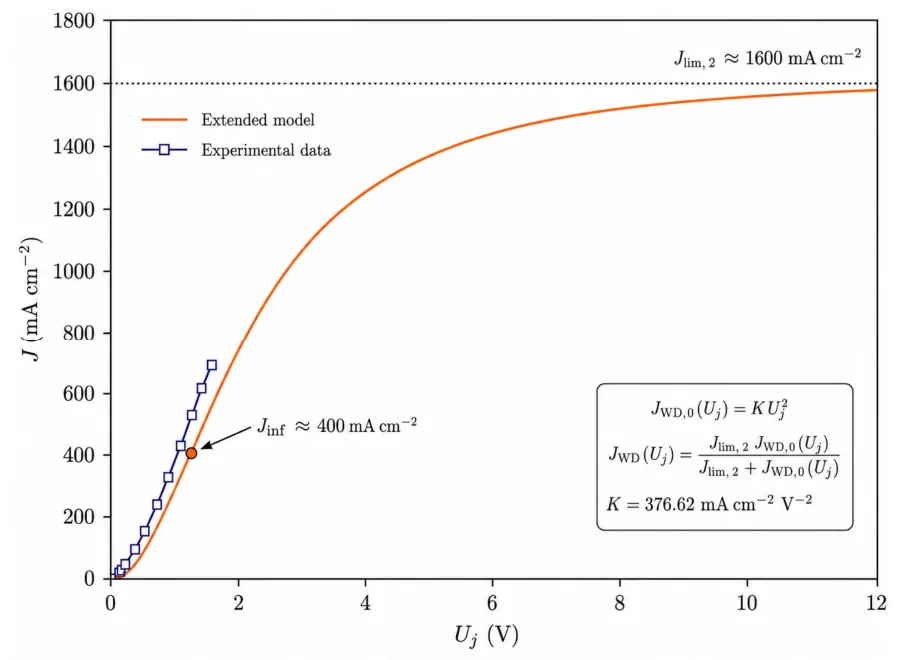
Saturation simulation for BPM-1 using $J_{lim,2}$ = 1600 mA cm^−2^.

**Figure 14:**
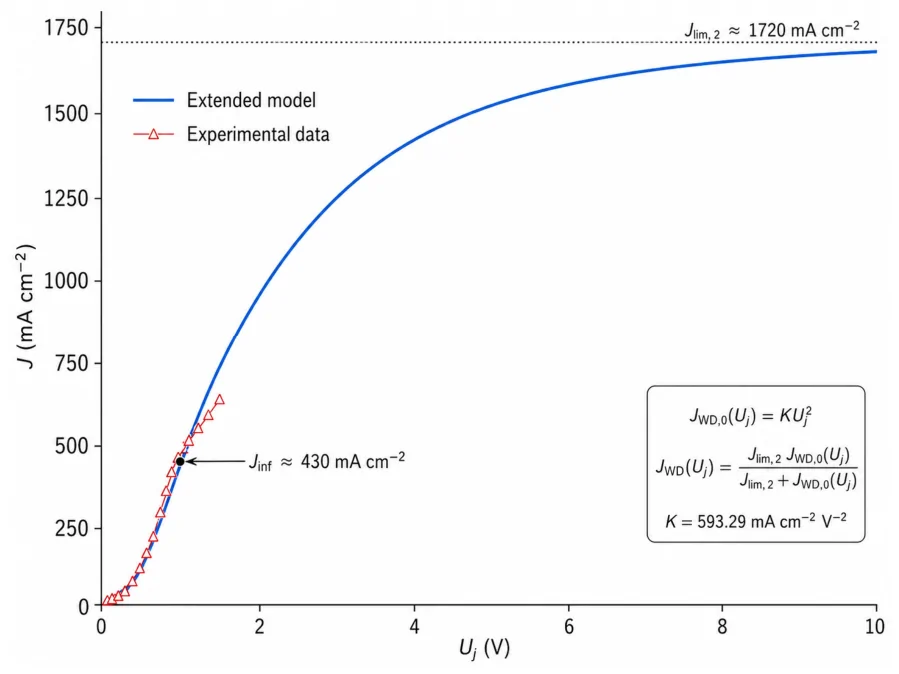
Saturation simulation for BPM-2 using $J_{lim,2}$ = 1720 mA cm^−2^.

The simulated saturation curves obtained using the experimentally fitted K values and Equation (37) reproduce the qualitative evolution of the high-current data (Figure 13 and Figure 14). They preserve the quadratic law at low and intermediate $U_{j}$, then progressively deviate toward a finite limiting current. Importantly, the experimentally observed deviation occurs well below the calculated $J_{lim,2}$, as predicted by the model. The inflection point corresponds to only 25% of the limiting current, meaning that a visible departure from the ideal quadratic law can occur long before the true plateau is reached. This result is conceptually important because it provides a compact analytical coupling between the intrinsic field-driven WD current and the high-current water-supply limitation. Previous studies have clearly identified water diffusion as a limiting process at high current density, and have treated it using detailed transport or multiphysics models [22,54,55], but the present formulation expresses the transition from $J_{WD,0}(U_{j})$ to $J_{lim,2}$ in a single saturation law.

To evaluate whether the estimated $J_{lim,2}$ values are physically reasonable, the corresponding effective water diffusion coefficients were calculated assuming approximately symmetric $D_{w,i}$, $L_{i}$, and $f_{w,i}$ for each layer i, where i refers to the AEL or CEL, and using the membrane data in Table 1. The parameters used in this estimate are summarized in Table 4.

The estimated effective diffusion coefficients are $D_{w,i}\;\approx \;6.6\;\times \;10^{-10}m^{2}\;s^{-1}$ for BPM-1 and $D_{w,i}\;\approx \;4.0\;\times \;10^{-10}m^{2}\;s^{-1}$ for BPM-2. These values are realistic for water confined in hydrated ion-exchange polymer domains [66]. Therefore, the $J_{lim,2}$ values inferred from the high-current deviation do not require unphysical water-transport parameters. This supports the interpretation that the departure from the ideal quadratic law is a physically plausible consequence of finite water supply to the BPM junction. Under the same symmetric approximation, $f_{eq}\;=\;f_{w}$, and the experimentally fitted K values correspond to effective junction thicknesses of approximately 0.88 nm for BPM-1 and 0.72 nm for BPM-2, which are consistent with a nanometric active WD region in homogeneous BPMs.

Overall, the high-current analysis extends the validation of the power-dissipation model beyond the purely quadratic regime. BPM-1 and BPM-2 first confirm the predicted $J\propto U_{j}^{2}$ dependence, then show a systematic downward deviation that can be rationalized by water-transport limitations. The relation $J_{inf}\;=\;0.25J_{lim,2}$ also provides a useful design criterion. It shows that the maximum sensitivity of the WD current to the junction voltage occurs at only one quarter of the water-supply-limited current. Therefore, increasing $J_{lim,2}$ is essential to extend the efficient WD regime to higher current density. From Equation (22), this requires thinner AELs/CELs, higher effective water availability $f_{w,i}$, and larger effective water diffusion coefficients $D_{w,i}$. Thus, BPM design should not only optimize field localization but must also ensure sufficient water transport to the junction.

## 4. Conclusions

This work provides an experimental assessment of the power-dissipation model for water dissociation in bipolar membranes. The results show that, once the WD-dominated region is isolated from the preceding mixed-transport regime, the polarization response of commercial BPMs can follow the predicted quadratic dependence on the effective junction voltage. This behavior was observed under both acid/base and neutral salt conditions, supporting the relevance of a field-driven interpretation of WD enhancement in depleted BPM junctions.

The study also shows that deviations from the quadratic law are physically informative rather than merely fitting failures. Catalyst-containing or heterogeneous interfaces can modify the apparent polarization response, while at high current density the finite supply of water to the junction leads naturally to saturation-type behavior. By coupling the intrinsic quadratic WD current to a water-transport limit, the extended model connects field-driven WD and high-current limitation within a single framework and predicts that the maximum sensitivity of the field-driven WD current occurs at only 25% of the second limiting current density. These results suggest that future BPM design should consider both the localization of the electric field at the junction and the ability of the AEL and CEL to sustain water transport under high-current operation.

## Figures and Tables

**Table 1:** Properties of the three investigated BPMs from suppliers and literature reports.

BPM Label	Product/Supplier Name	Type	Interfacial Catalyst	Thickness (µm)	Water Content
BPM-1	Alfa Chemistry	Homogeneous (supplier)	-	200–220 (supplier)	20–30% (supplier)
BPM-2	Fumasep FBM	Homogeneous [56]	-	130–160 (supplier)	~32.5% from [57]
BPM-3	Neosepta BPU	Homogeneous [1]	Yes, reported in [58]; generally metal hydroxide-based catalyst [59]	280 (supplier)	Not disclosed; not considered in this study

**Table 2:** Quadratic fitting parameters of the acid/base polarization curves of the three BPMs.

Membrane	$U_{oc}$ Used for $U_{j}$ (V)	$J_{t}$ (mA cm^−2^)	K (mA cm^−2^ V^−2^)	R^2^
BPM-1	0.805	20.75	398.94	0.9994
BPM-2	0.819	17.80	556.14	0.9890
BPM-3	0.814	100.60	623.33	0.9816

**Table 3:** Quadratic fitting parameters of neutral salt polarization curves of the three BPMs.

Membrane	$U_{wd}$ (V)	K (mA cm^−2^ V^−2^)	R^2^
BPM-1	0.61	240.45	0.9999
BPM-2	0.80	435.43	0.9978
BPM-3	0.63	642.47	0.9991

**Table 4:** High-current model parameters for BPM-1 and BPM-2.

Parameter	BPM-1	BPM-2
K (mA cm^−2^ V^−2^)	376.62	593.29
$J_{WD,inf}$ (mA cm^−2^)	400	430
$J_{lim,2}$(mA cm^−2^)	1600	1720
$L_{i}$ (μm)	110	80
$f_{w,i}$	0.25	0.325
$f_{eq}$	0.25	0.325
Estimated $D_{w,i}$ (m^2^ s^−1^)	6.6 × 10^−10^	4 × 10^−10^
Estimated δ (nm)	0.88	0.72

## Data Availability

The original contributions presented in this study are included in the article. Further inquiries can be directed to the corresponding author.

## Abbreviations

A	Compact water-supply parameter, A = ($D_{w,AEL}/L_{AEL})f_{w,AEL}+\left(D_{w,CEL}/L_{CEL}\right)f_{w,CEL}$
B	Compact water-supply conductance parameter, ($B=D_{w,AEL}/L_{AEL}+D_{w,CEL}/L_{CEL}$)
$c_{ions}$	Concentration of autoprotolysis ions $H_{3}O^{+}$ and $OH^{-}$ in water in mol·L^−1^
$c_{H_{2}O,l}$	Concentration of free liquid water in mol·L^−1^ (≈55.5 at 25 °C)
$c_{H_{2}O,j}$	Water concentration in the BPM junction in mol·L^−1^
$c_{H_{2}O,i}^{outer}$	Water concentration at the outer interface of membrane layer (i) in mol·L^−1^
$D_{w,i}$	Water diffusion coefficient in membrane layer (i) in m^2^s^−1^
E	Local electric field in the BPM junction in V·m^−1^
$E_{d}$	Threshold dissociation energy per water molecule in Joules, J
*f*	Effective water availability or hydration factor in the junction ($f=c_{H_{2}O,j}/c_{H_{2}O,l}$)
$f_{eq}$	Conductance-weighted equilibrium water fraction supplied by the AEL and CEL
($f_{w,i}$)	Water fraction at the outer side of the membrane layer (i); dimensionless
F	Faraday constant 96,485 C·mol^−1^
i	Membrane layer index, i = AEL or CEL
J	Current density in mA·cm^−2^
$\bar{J}$	Mean current density over the fitted region in mA·cm^−2^
$J_{i}$	Experimental current density at data point (i) in mA·cm^−2^
$J_{i,fit}$	Fitted current density at data point (i) in mA·cm^−2^
$J_{t}$	Transition current density separating the mixed-current region from the WD-dominated region in mA·cm^−2^
$J\left(U_{wd}\right)$	Current density at the effective WD transition voltage in neutral pH experiments in mA·cm^−2^
$J_{WD}$	Water-dissociation current density in mA·cm^−2^
$J_{WD,0}\left(U_{j}\right)$	Intrinsic field-driven WD current density in the absence of water-transport limitations in mA·cm^−2^
$J_{WD,inf}$	WD current density at the inflection point of the saturation curve in mA·cm^−2^
$J_{lim,2}$	Water-supply-limited current density, also referred to as the second limiting current density in mA·cm^−2^
$J_{w,i}$	Diffusive water flux through membrane layer (i) in mol·m^−2^· s^−1^
*K*	Apparent quadratic prefactor of the WD current–voltage law in mA·cm^−2^·V^−2^
$K_{WD}$	Quadratic coefficient excluding the water-availability factor in mA·cm^−2^·V^−2^
$k_{d}\left(E\right)$	Field-enhanced water-dissociation rate constant in s^−1^
$L_{i}$	Thickness of membrane layer (i) in meters (m)
$U_{BPM}$	BPM voltage measured between the two reference electrodes in Volts (V)
$U_{eq}$	Equilibrium BPM voltage associated with the imposed pH gradient in V
$U_{oc}$	Open-circuit or near-zero-current BPM voltage in V
$U_{j}$	Effective junction driving voltage localized in the water-dissociation region in V
$U_{wd}$	Effective transition voltage above which the neutral pH response becomes WD-dominated in V
δ	Effective thickness of the active BPM junction in m
$\eta_{WD}$	Water-dissociation overpotential in V
μ	Ionic mobility in m^2^·V^−1^·s^−1^
x	Spatial coordinate across the membrane layers and BPM junction
$x_{j}$	Position of the BPM junction
$x_{L}$	Position of the left outer membrane interface
$x_{R}$	Position of the right outer membrane interface
